# Enhancing Skin Permeation of Biphenylacetic Acid (BPA) Using Salt Formation with Organic and Alkali Metal Bases

**DOI:** 10.3797/scipharm.1406-19

**Published:** 2014-09-26

**Authors:** Vijay Pawar, Prashant Naik, Rajani Giridhar, Mange Ram Yadav

**Affiliations:** Pharmacy Department, Faculty of Technology & Engineering, The M. S. University of Baroda, Vadodara-390001, India

**Keywords:** BPA, Lipophilicity, NSAID, Salt, Skin permeation

## Abstract

In the present study, a series of organic and alkali metal salts of biphenylacetic acid (BPA) have been prepared and evaluated *in vitro* for percutaneous drug delivery. The physicochemical properties of BPA salts were determined using solubility measurements, DSC, and IR. The DSC thermogram and FTIR spectra confirmed the salt formation with organic and alkali metal bases. Among the series, salts with organic amines (ethanolamine, diethanolamine, triethanolamine, and diethylamine) had lowered melting points while the alkali metal salt (sodium) had a higher melting point than BPA. The *in vitro* study showed that salt formation improves the physicochemical properties of BPA, leading to improved permeability through the skin. Amongst all the prepared salts, ethanolamine salt (**1b**) showed 7.2- and 5.4-fold higher skin permeation than the parent drug at pH 7.4 and 5.0, respectively, using rat skin.

## Introduction

Arthritis is a form of joint disorder involving inflammation, pain, and swelling of one or more joints. NSAIDs are commonly used to treat various forms of arthritis such as rheumatoid arthritis (RA) and osteoarthritis (OA). However, long-term systemic use of NSAIDs is often limited by a wide range of adverse events. The extensive use of oral NSAIDs results in gastrointestinal, cardiovascular, and renal adverse effects [1–5]. In rheumatic disorders, the percutaneous delivery of drugs to the underlying muscle and joint tissues is of considerable importance. Hence, a potential way to mitigate the side effects of orally administered NSAIDs is their topical application [6]. The clinical efficacy of a topical NSAID preparation has been confirmed in patients with a variety of painful disorders [7]. Pharmacokinetic data of commercially available diclofenac sodium 1% gel (Voltaren; Novartis) show that compared to the oral diclofenac, topical diclofenac sodium (1% gel) produces lower mean plasma concentration and minimizes the inherent side effects [8].

Percutaneous drug delivery allows the localization of the drug at the target site [9]. The rate of transport across the skin depends on both the nature of the solute and the nature of the vehicle [10]. However, due to the unfavorable physicochemical properties of NSAIDs and the hindrance posed by skin barriers, the majority of the topically applied NSAIDs have low permeability through human skin [11]. The main driving force for the development of topical agents has been the potential avoidance of adverse reactions associated with systemic agents, increased concentration of the active moiety at the site of application, and other advantages such as the ease of application and termination of the therapy. Studies have confirmed that most of the drug present in the muscle and skin is due to direct migration and not from systemic circulation following topical application [6, 12].

Various strategies have been developed and used to improve the percutaneous delivery of NSAIDs that include the formulation approach, pro-drug approach, and salt formation [13–15]. In the formulation approach, penetration enhancers have been used to improve skin permeability. These penetration enhancers partition into the skin, interact with the constituents of the skin layers, and reduce the resistance of the skin to drug diffusion [16, 17]. They may cause skin irritation and other side effects. The pro-drug approach has been used increasingly to improve the delivery of drugs through the skin and involves the modification of the basic structure of a drug; however, a high pro-drug concentration in the skin may lead to enzyme saturation kinetics and as a result, limited conversion of the pro-drug to the parent drug might occur [18–21].

Salts are usually considered as alternatives for the delivery of a drug when the parent drug molecule is unsuitable for a formulation. This strategy enhances the transportation of the ionic drugs through the skin without modifying the molecular structure of the drug [22, 23]. It has been observed that biphenylacetic acid (BPA) has severe GIT side effects with oral administration due to its free carboxylic acid group that restricts its oral use [24]. Studies demonstrated that plasma levels of BPA following topical application were low and well below the reported therapeutic concentration of BPA [25]. This indicates that BPA is slowly absorbed into the bloodstream and has low permeability through the skin. The choice of the most appropriate drug for percutaneous delivery depends on a number of factors that include its potency, half-life, permeability, lack of local skin toxicity, etc. [26, 27]. In a recent study, nine commercially available topical NSAID preparations from the European Union have been studied for skin permeability, anti-inflammatory, and analgesic actions. This study revealed that the topical ketoprofen preparation showed superior results as compared to the other preparations [28]. From this study we can conclude that along with permeation enhancement, the pharmacokinetic and pharmacodynamic properties of NSAIDs are also important parameters from the therapeutic point of view for rheumatic diseases. For the safe and effective treatment of osteoarthritis (OA), the current new guidelines also recommended the use of topical NSAIDs, but the majority of the clinically used NSAIDs have short half-lives, low permeability, and lack affinity to the joint cavity which adversely affects the treatment of rheumatic diseases [8].

In the present study we have prepared various salts of BPA, an active metabolite of fenbufen, which is twice as active as the parent drug, having a long half-life (>10 h) and proven to be compatible and safe for topical application [25, 29]. Based on these favorable pharmacokinetic and pharmacodynamic properties, we synthesized and evaluated the salts of BPA to improve the skin permeability of BPA. For the synthesis of salts, various organic and alkali metal bases such as ethanolamine, diethanolamine, triethanolamine, diethylamine, and sodium hydroxide have been used and the differences between the physicochemical characteristics of BPA and its salts have been evaluated by using DSC, FTIR, SEM, etc. The effect of salt formation on percutaneous absorption has been investigated *in vitro* using skin from the whole dorsal area of a male Wistar rat.

## Results and Discussion

### Syntheses of BPA Salts

**Fig. 1:**
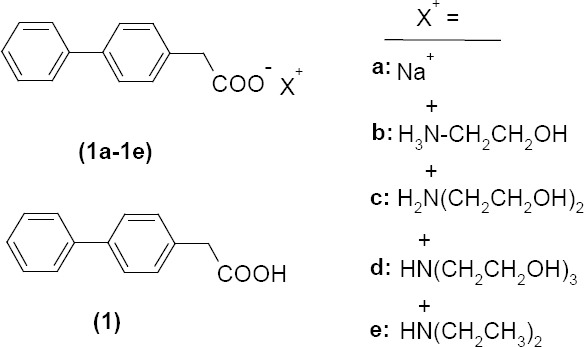
The structures of BPA (**1**) and its salts (**1a–e**)

BPA (**1**), required for the synthesis of the salts, was prepared by the reported procedure [31]. In the current study, due to the presence of the acidic functional group in BPA, we prepared some salts of this NSAID with organic and alkali metal bases. Alkanolamines such as ethanolamine, diethanolamine, triethanolamine, and diethylamine (DEA) have been chosen for this purpose because these alkanolamines are weak bases. The amino group will react with the acidic carboxylic acid group present in the BPA resulting in salt formation. Structures of the prepared salts are shown in Fig. 1.

### Characterization of Salts

The prepared salts have been confirmed by various techniques such as FTIR, NMR, DSC, and SEM, which have been used to characterize the surface morphology of the prepared salts. The physicochemical and spectral data of the compounds are listed in Table 1.

**Tab. 1:**
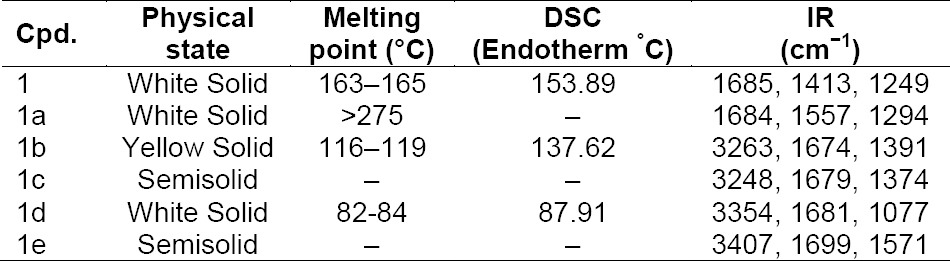
Physicochemical and spectral data of compounds (**1, 1a–e**)

**Fig. 2:**
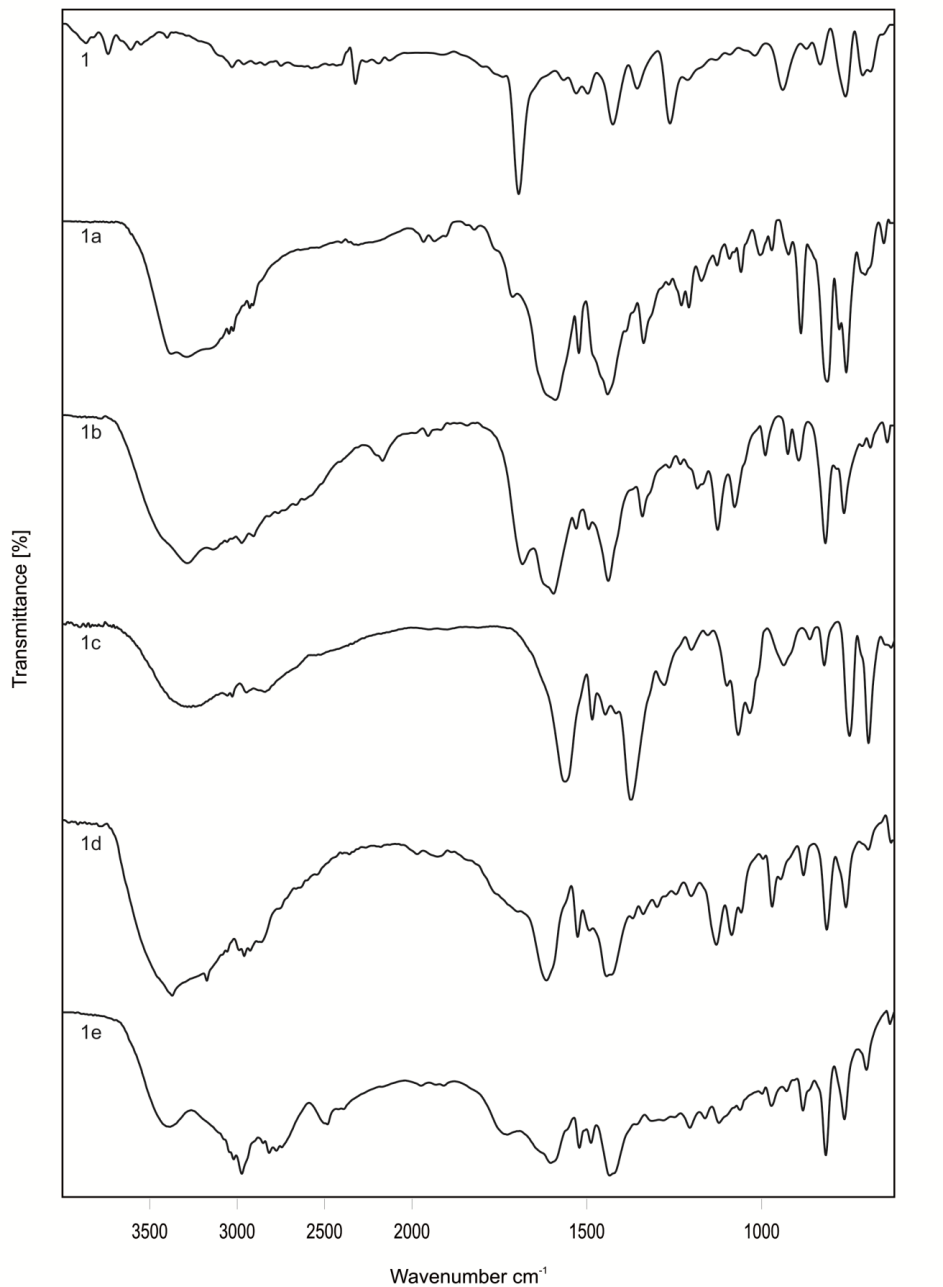
FTIR Spectra of BPA (**1**) and its salts (**1a–e**)

The prepared salts were characterized by using IR. The FTIR spectra of BPA (**1**) and its salts are shown in Fig. 2. As expected, the obvious difference between the spectra of BPA and its salts was found in the O-H and N-H stretching regions. Alkanolamine salts showed peaks in the range of 3263–3404 cm^−1^ due to N-H stretchings which were absent in BPA (**1**). Other signs of interactions were reflected by the presence of a strong sharp signal around 1685 cm^−1^, characteristic of the carbonyl stretching vibrations. The carbonyl peak of the BPA in the salts was shifted due to the interaction between the negative charge on the oxygen of BPA (**1**) and the positive charge on the nitrogen atom of alkanolamines.

In the NMR spectrum, aromatic protons of BPA appeared at δ 7.88-7.28 as a multiplet, methylene protons at δ 3.65 as a singlet, and the acidic proton at δ 8.84. In the case of the ethanolamine salt of BPA, the obvious difference was the presence of additional peaks at δ 3.62–3.60 and at δ 3.22–3.18 as triplets for methylene protons of ethanolamine and a broad peak at δ 6.07 due to the protons of the ammonium group. Fig. 4. shows DSC curves of BPA (**1**) and its salts (**1a–e**). Table 1 summarizes the melting points and endothermic peaks of BPA (**1**) and its salts (**1a–e**). The melting points of the salts decreased remarkably compared to the parent drug except for the sodium salt (**1a**) which showed a higher melting point. Shifts in melting points are indicative of salt formation.

**Fig. 3:**
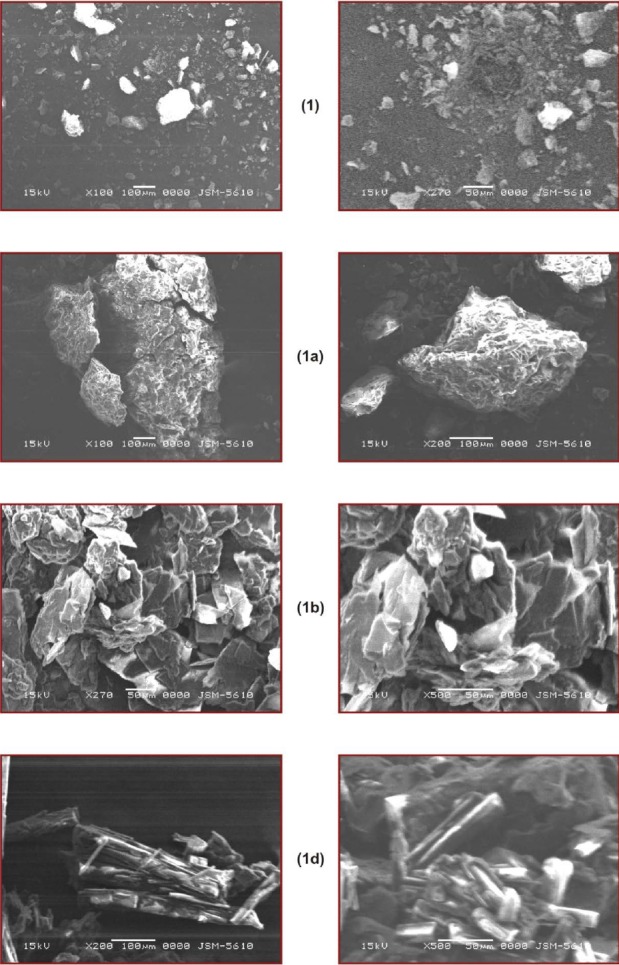
Scanning electron microscope images of compounds (**1, 1a, 1b, 1d**)

**Fig. 4:**
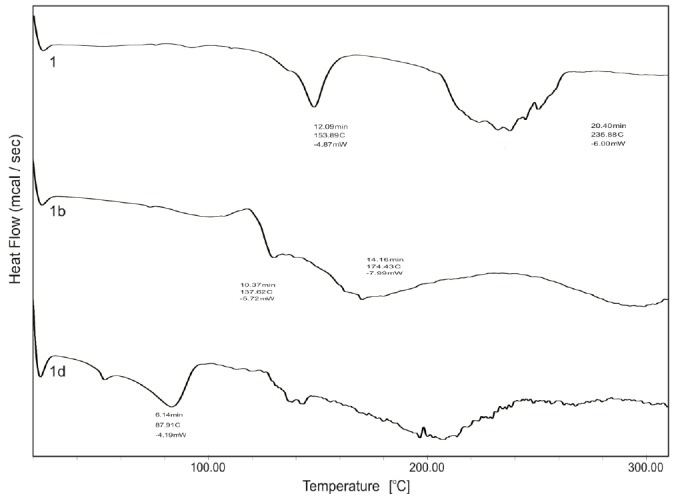
DSC thermograms of BPA (**1**) and its salts (**1b, 1d**)

The surface morphology of the compounds varied from each other and to evaluate this parameter, the SEM of the prepared salts was performed. From this study, it was concluded that each salt has a different surface morphology as shown in Fig. 3.

### Physicochemical Evaluation

For the evaluation of various physicochemical parameters such as aqueous solubility, partition coefficient, and *in vitro* skin permeability, the HPLC method was developed, calibration curves for BPA (**1**) were plotted, and the linearity range was calculated.

### Determination of Solubility in Phosphate Buffer

**Fig. 5:**
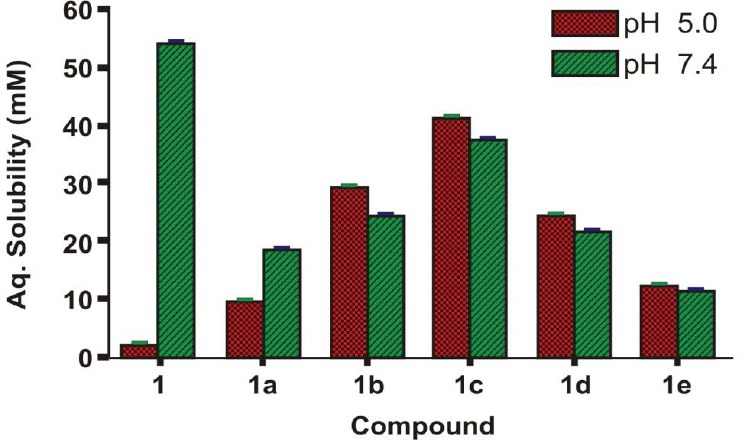
Aqueous solubility of BPA (**1**) and its salts (**1a–e**)

Due to the biphasic nature of the skin, the ideal salt form should exhibit adequate lipid solubility as well as aqueous solubility. Aqueous solubility of BPA and its salts was determined in a phosphate buffer (0.16 M) at the physiological pH 5.0 and at pH 7.4 as the environment of the outer surface of the skin is acidic (pH 4.2–6.5) [11]; the values are given in Table 2. Fig. 5. shows the aqueous solubility of BPA (**1**) and its salts (**1a-1e**).

**Tab. 2:**
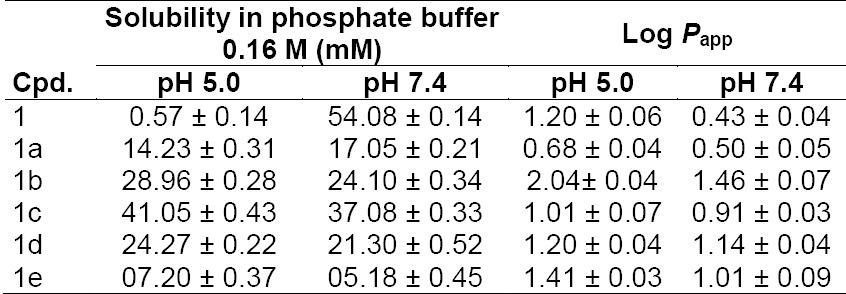
Solubility and partition coefficient (Log *P*_app_) values of BPA (**1**) and its salts (**1a–e**) at pH 5.0 and pH 7.4

### Determination of the Apparent Partition Coefficient (Log P_app_)

**Fig. 6:**
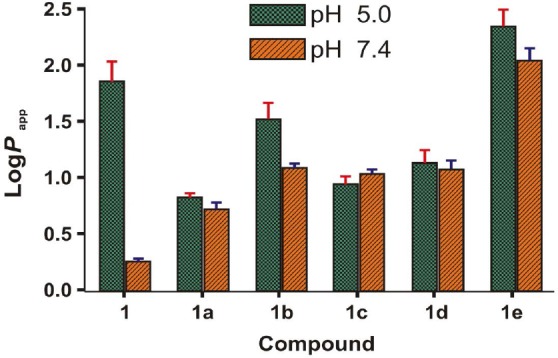
Partition coefficient (Log *P*_app_) values of BPA (**1**) and its salts (**1a–e**)

Lipid solubility plays a crucial role in determining the skin permeability of a compound because the SC (stratum corneum), the major barrier to drug permeation, is essentially lipoidal in nature and generally favors the permeation of lipophilic drugs [11]. The apparent partition coefficients of BPA (**1**) and salts (**1a–e**) were determined by partitioning them between the phosphate buffer (0.16 M) and 1-Octanol at both pH 5.0 and pH 7.4 by the shake flask method and are shown in Fig. 6. Table 2 shows the Log *P*_app_ values of BPA (**1**) and its salts (**1a–e**).

### *In Vitro* Skin Permeation Study

**Fig. 7:**
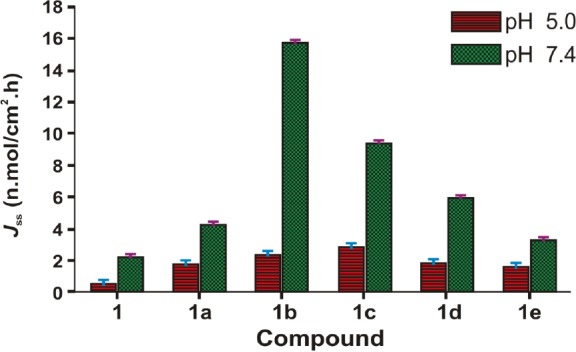
Steady-state flux (*J*_ss_) of 6-MNA (**1**) and the salts (**1a–e**) through rat skin *in vitro* at 37°C

**Fig. 8:**
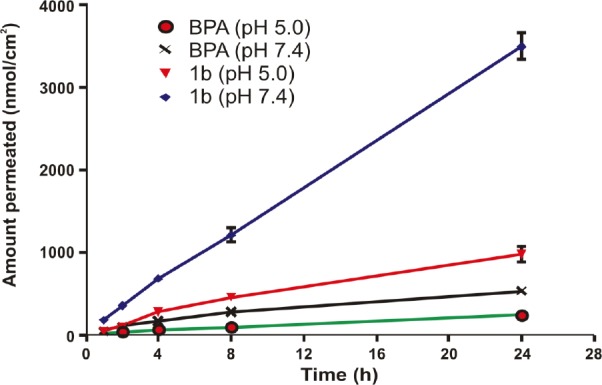
Permeation profile of BPA (**1**) and its salt (**1b**) (mean±SEM, n=3-6) at pH 5.0 and 7.4

The *in vitro* skin permeation study was performed by using skin from the whole dorsal area of a male Wistar rat. The *in vitro* diffusion experiments showed that the salts of BPA were able to permeate skin. For each salt and the parent drug, the cumulative amounts permeated through the skin were plotted against time. A steady-state flux (*J*ss) was obtained by dividing the slope of that graph by the surface area of the diffusion cell (4.906 cm^2^) [11]. The steady-state flux (*J*ss) of BPA (**1**) and its salts (**1a–e**) is given in Table 3. and shown in Fig. 7. All the salts have shown higher flux values than the parent NSAID and this is due to ion-pair formation. Amongst all the salts, **1b** has shown the highest steady-state flux including BPA. The permeation profiles of BPA (**1**) and its salt (**1b**) are shown in Fig. 8.

**Tab. 3:**
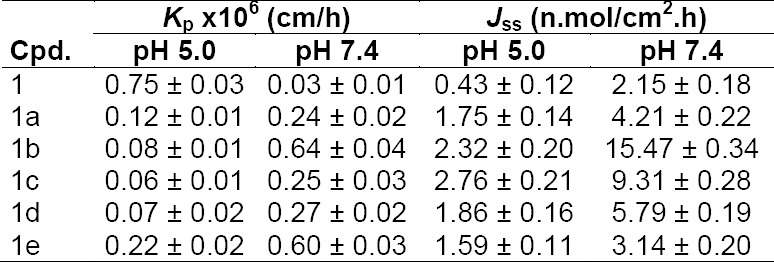
Steady-state flux (*J*_ss_) and permeability coefficients (*K*_p_) of BPA (1) and its salts (1a–e)

The ethanolamine salt displayed 7.2-times higher flux than the parent drug. The result also showed that the salts with higher flux have a balance between aqueous solubility and the partition coefficient. Further, except for the sodium salt, all the salts have shown lower melting points than the parent drug and higher permeability through the skin, which support previous reports, indicating that a decrease in melting point or conversion of solid state to liquid state improves the permeability of the drugs through the skin [12]. Further, we can conclude that the significant enhancement of the skin permeability of BPA is the result of salt formation or ion pairing which is in good agreement with previously published work [22, 23, 30].

## Experimental

All the reagents and solvents required for the syntheses were purified by general laboratory techniques before use. The purity of the compounds and completion of the reactions were monitored by thin layer chromatography (TLC) on silica gel plates (60 F_254_; Merck), visualized with ultraviolet light or iodine vapors. The yields reported here are un-optimized. Melting points were determined using a Veego make silicon oil bath-type melting point apparatus and are uncorrected. The IR spectra were recorded using the KBr disc method in cm^−1^ on a Bruker FTIR, Model 8300. The PMR spectra were recorded in CDCl_3_ on a Bruker 400 MHz spectrometer (chemical shifts in δ ppm, coupling constant *J* in Hz). *λ*_max_ was determined on the Shimadzu 1800 UV spectrophotometer. The mass of the compounds was determined by LC-MS using electron impact as the source of ionization. DSC was performed on the Shimadzu DSC-60 model with the thermal analyser TA 60WS. The scanning electron microscope (SEM) model ESEM-EDAX XL-30, Philips (Netherlands) was used for the study of surface morphology. *In vitro* skin permeation studies were conducted using a Franz-type diffusion cell using skin from the whole dorsal area of a male Wistar rat (200 ± 10 g). Animal experiments were performed with the approval of the institutional animal ethical committee (MSU/PHARM/IAEC/2011/14) in accordance with Laboratory Animal Welfare guidelines.

### Synthesis of Salts

BPA (**1**), required for the synthesis of the salts, was prepared by the reported procedure [31]. BPA (**1**) was dissolved in dichloromethane with the help of a small amount of methanol to make the solution clear and an equimolar amount of base (alkanolamines or sodium hydroxide) was added and the reaction mixture was stirred for 5–6 h. The precipitated salt was collected by filtration and recrystallized from ethyl acetate to yield pure salts (**1a–e**) of BPA.

### HPLC Analysis

HPLC analysis was performed by using the Shimadzu Prominence UV/VIS (Pump LC-20AT, detector SPD 20 A) using the column Purospher 5 μm (e) C-18, 4.6 × 250 mm and column temperature 25–28°C. Chromatography was performed under isocratic conditions, at a flow-rate of 1 ml/min. The mobile phase consisted of a phosphate buffer (PB, 15 mM)-acetonitrile of pH 5.0–5.5. The injection volume was 20 μl and and the detection wavelength was 228.0 nm. The calibration plot and linearity were determined, and the retention time of BPA was found to be 3.0 min. The limits of detection for the analytes were between 0.122 and 0.468 μg ml^−1^ and the limits of quantification were between 0.360 and 1.418 μg ml^−1^.

### Solubility in Phosphate Buffer (0.16 M)

The aqueous solubility of BPA (**1**) and its salts (**1a–e**) was determined in the phosphate buffer (0.16 M) at both pH 5.0 and 7.4 at room temperature. Excess amounts of each salt were added to the phosphate buffer (0.5 ml) individually. The mixtures were stirred and centrifuged at 6000 rpm for 6 min. The concentrations of each of the salts in their saturated solutions were analyzed by HPLC. The pH of the solutions was held constant throughout the experiment [11].

### Apparent Partition Coefficient (Log P_app_)

The apparent partition coefficients (log *P*_app_) of BPA (**1**) and its salts (**1a–e**) were determined at room temperature between 1-Octanol and the phosphate buffer (0.16 M) at pH 5.0 and 7.4 using the shake flask method. 1-Octanol was saturated with the phosphate buffer for 24 h by stirring vigorously before use. A known concentration of the compound in the phosphate buffer was shaken with a suitable fixed volume of 1-Octanol. After shaking, both the phases were separated by centrifugation at 6000 rpm for 6 min. The concentrations of the compounds in the buffer phase and in the 1-Octanol phase were determined by HPLC [11].

### In Vitro Skin Permeation Studies

*In vitro* skin permeation studies were performed by using skin from the whole dorsal area of a male Wistar rat in a Franz-type diffusion cell. Skin specimens were rehydrated before being mounted in the diffusion cell. The receptor medium (0.05 M phosphate buffer saline solution of pH 7.4) was stirred and kept at 37±1°C throughout the study. The compounds (**1a-1e**) were applied as solutions in the phosphate buffer (0.05 M) at both pH 5.0 and 7.4.

The steady-state flux for BPA (**1**) and its salts (**1a–e**) were determined by plotting the cumulative amount of the drug as measured in the receiver phase against time, and dividing the slope of the steady-state position by the surface area of the diffusion cell (4.906 cm^2^). The permeability coefficients (*K*_p_) for the steady-state delivery were obtained by dividing the steady-state flux (*J*_ss_) by the solubilities of the compounds in the corresponding vehicle [11]. The receiver medium (0.05 M phosphate buffer saline solution of pH 7.4) was stirred and kept at 37±1°C throughout the study. The compounds were applied as solutions in the phosphate buffer of pH 5.0 or 7.4. At specified time intervals, aliquots (0.5 ml) were withdrawn from the receiver compartment and replaced with fresh buffer. The drug concentrations were analyzed by HPLC.

## Conclusion

In conclusion, the present study shows that the permeation of BPA through rat skin can be markedly improved by the salt formation approach. Salt formation improves the aqueous solubility and other physicochemical properties essential for drug permeation, and these improvements make these salts promising moieties for the percutaneous delivery of BPA. Increased permeation of BPA coupled with a long half-life and slow clearance from the body can increase local tissue and synovial fluid concentration which is essential for symptomatic relief of rheumatic diseases.
